# Snailase Preparation of Ginsenoside M1 from Protopanaxadiol-Type Ginsenoside and Their Protective Effects Against CCl_4_-Induced Chronic Hepatotoxicity in Mice

**DOI:** 10.3390/molecules161210093

**Published:** 2011-12-06

**Authors:** Wei Li, Ming Zhang, Yi-Nan Zheng, Jing Li, Ying-Ping Wang, Yun-Jing Wang, Jian Gu, Ying Jin, Hui Wang, Li Chen

**Affiliations:** 1 Norman Bethune College of Medicine, Jilin University, Changchun 130021, China; 2 College of Chinese Medicinal Materials, Jilin Agricultural University, Changchun 130118, China; 3 Institute of Special Wild Economic Animals and Plant, CAAS, Jilin 132109, China; 4 China-Japan Union Hospital, Jilin University, Changchun 130033, China

**Keywords:** ginsenoside M1, protopanaxadiol-type ginsenoside, hepatoprotective effect

## Abstract

To investigate the protective effects of protopanaxadiol-type ginsenoside (PDG) and its metabolite ginsenoside M1 (G-M1) on carbon tetrachloride (CCl_4_)-induced chronic liver injury in ICR mice, we carried out conversion of protopanaxadiol-type ginsenosides to ginsenoside M1 using snailase. The optimum time for the conversion was 24 h at a constant pH of 4.5 and an optimum temperature of 50 °C. The transformation products were identified by high-performance liquid chromatography and electrospray ion-mass spectrometry. Subsequently, most of PDG was decomposed and converted into G-M1 by 24 h post-reaction. During the study on hepatoprotective in a mice model of chronic liver injury, PDG or G-M1 supplement significantly ameliorated the CCl_4_-induced liver lesions, lowered the serum levels of select hepatic enzyme markers (alanine aminotransferase, ALT, and aspartate aminotransferase, AST) and malondialdehyde and increased the activity of superoxide dismutase in liver. Histopathology of the liver tissues showed that PDG and G-M1 attenuated the hepatocellular necrosis and led to reduction of inflammatory cell infiltration. Therefore, the results of this study show that PDG and G-M1 can be proposed to protect the liver against CCl_4_-induced oxidative injury in mice, and the hepatoprotective effect might be attributed to amelioration of oxidative stress.

## 1. Introduction

The liver plays a key role in the metabolism, detoxification, and secretionary functions of the body. Hepatic injury is a fundamental pathological process in most chronic hepatic diseases and long-standing hepatic injury leads to the progressive liver injury, fibrosis, and finally cirrhosis [1]. As it is well known, a wide variety of viruses, drugs and toxic chemicals can cause liver injury by means of their direct toxicity and/or endogenous toxic metabolic products [2,3,4]. Carbon tetrachloride (CCl_4_) is a chemical hepatotoxin, which is considered the “gold standard” model of hepatic injury that has the advantage of mimicking that caused by different etiologies in humans [5,6]. Extensive research over the past decades indicates that some herbal extracts and their chemical constituents can significantly inhibit these aforementioned pathologic processes and provide protection against acute and chronic liver damage [7,8,9].

**Figure 1:**
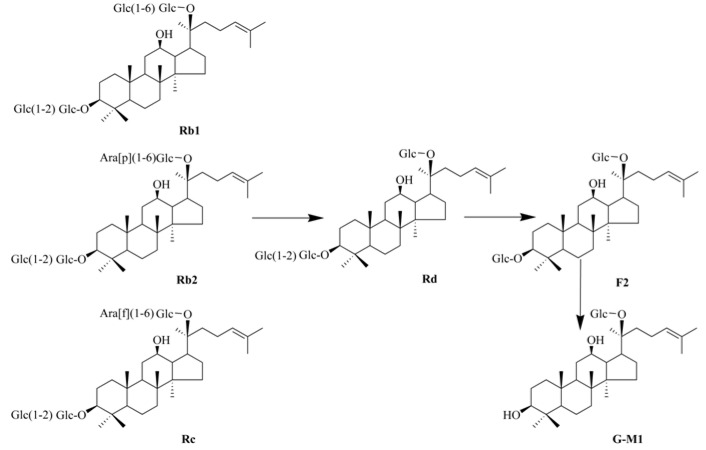
Proposed bioconversion pathway of PDG to G-M1.

Ginseng, the root of *Panax ginseng* C. A. Meyer, is one of the most famous herbal medicines in China, Japan, Korea and other Asian countries [10]. Generally, the pharmacological properties of ginseng are attributed to its components the ginsenosides, which can be classified into protopanaxadiol-type ginsenosides (PDG, e.g., ginsenosides Rb1, Rb2, Rc, Rd, Rg3, Rh2) and protopanaxatriol-type ginsenosides (PTG, e.g., ginsenosides Re, Rg1) based on their sapogenins [11]. It is well known that PDG are metabolized by intestinal bacteria after oral administration to their final derivative 20-*O*-β-D-glucopyranosyl-20(*S*)-protopanaxadiol (ginsenoside M1, in Figure 1) [12,13]. Recently, G-M1 has attracted increasing attention in view of its various biological activities, including anti-cancer [14,15], anti-inflammation [16] and anti-diabetes effects [17,18].

In the past several years, various transformation methods, including mild acid hydrolysis [19] and microbial conversion [20,21], were used to convert the main ginsenosides to G-M1. However, these transformation methods were time-consuming, and resulted in low selectivity and low conversion rates. As an alternative of the above preparation methods, enzymatic preparation (EP) has been proposed as the most promising for the preparation of active constituents via the selective hydrolysis of the sugar moieties, owing to its high specificity, yield and productivity. Recently, snailase (a complex of cellulase, hemicellulase, pectinase and β-glucuronidase), extracted from the digestive tract of snails [22], has received increasing attention due to its strong hydrolytic abilities [23]. During our research aiming to convert main protopanaxadiol-type ginsenosides to ginsenoside M1, we employed snailase to transform PDG to GM1 with great success after its comparing hydrolysis ability with other conventional chemical enzymes (Figure 1 shows the proposed bioconversion pathway of PDG to G-M1).

Furthermore, though the hepatoprotective effects of ginsenosides Rb1 and G-M1 in *tert-*butyl hydroperoxide-induced acute hepatotoxicity have been reported, little attention has been focused on comparison of PDG and G-M1 for treating chronic liver injury. Park *et al*. have reported that G-M1 induces apoptosis in T-HSC/Cl-6 cells via caspase-3 activation [24]. To accomplish this goal, we transformed PDG by snailase to G-M1, and a classic CCl_4_-induced chronic liver injury model was chosen to study the liver protective effects of PDG and G-M1 in mice. This should be helpful for understanding the effect relationship between PDG and G-M1, providing a scientific basis for their application as a dietary supplement or drug for the treatment of hepatic injury.

## 2. Results and Discussion

### 2.1. Biotransformation of PDG to G-M1

**Figure 2:**
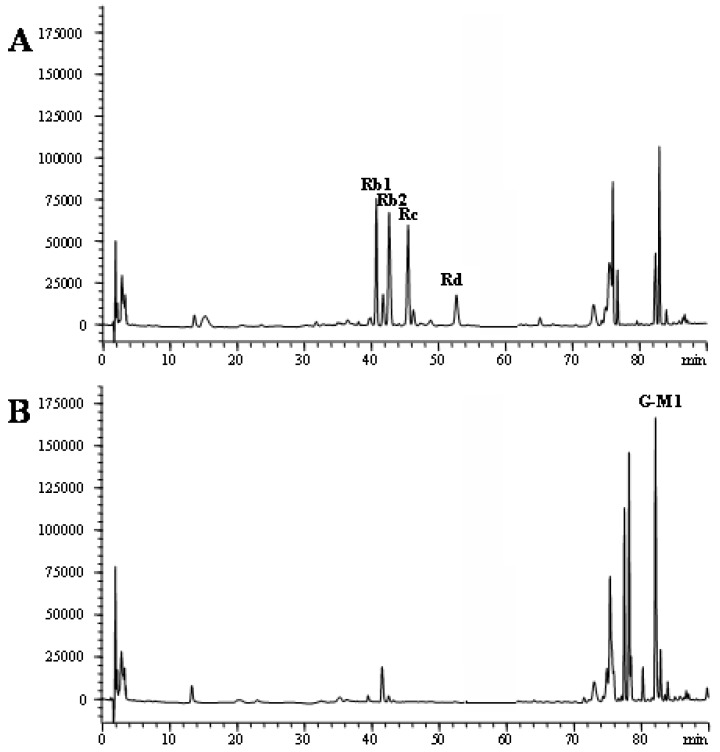
HPLC analysis of the bioconversion of protopanaxadiol-type ginsenosides to G-M1. Incubation time: A, 0 h, B, 24 h.

PDG was isolated from ginseng roots by the previously reported method [25]. PDG is mainly composed of ginsenosides Rb1, Rb2, Rc, and Rd with contents of 221 mg/g, 198 mg/g, 178 mg/g and 55 mg/g, respectively. The hydrolyzing ability to convert PDG to CK of several glycolytic enzymes based on the glycosidic moiety, snailase, β-glucanase, cellulase and amylase, were evaluated. Although the above enzymes gave the same hydrolysis pattern and similar ability, complete hydrolysis to PDG in 24 h was only achieved by snailase. Therefore snailase was used to convert PDG to G-M1 under optimized conditions. In brief, the snailase was incubated with PDG in a pH 4.5 sodium acetate buffer with agitation at a temperature of 50 °C for a reaction time of 24 h. The mixtures were subsequently placed in a water bath at 90 °C to finish the enzymatic reaction. The reaction mixtures were individually evaporated, dissolved in methanol, and loaded onto a silica gel column (80 cm × 3 cm, I.D.; solvent, CHCl_3_-MeOH = 20:1–15:1) to give G-M1. Figure 2 shows the chromatograms of PDG before and after enzymatic preparation.

### 2.2. Identification of G-M1 by ESI-MS

**Figure 3:**
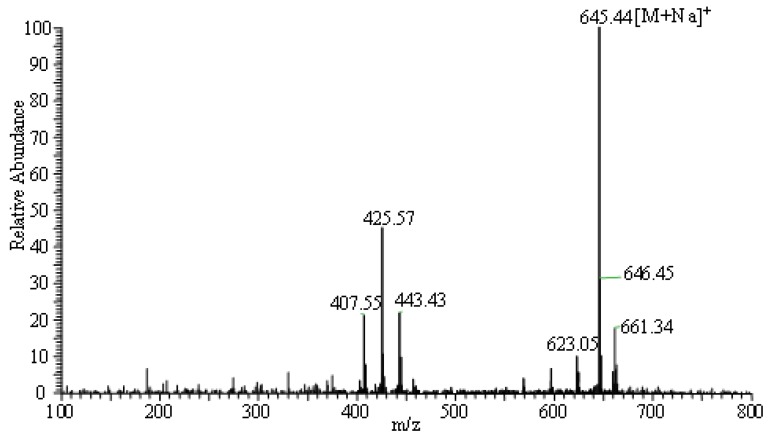
ESI/MS spectra of G-M1. Total ion chromatograms from 100 to 800 m/z in ESI positive mode.

Mass spectrometry (MS), especially MS with electrospray ionization (ESI-MS), is a valuable analytical tool in term of providing information on the molecular weights of polar and thermally labile compounds. In the present study, the structure of G-M1 was confirmed by ESI/MS. As shown in Figure 3, a high abundance of [M+Na]^+^ (645.44) is observed in the positive mode. The results showed that the molecular weight of G-M1 was 622.

### 2.3. Effects of PDG and G-M1 on Body and Organ Weights

As Table 1 shows, body weights of the experimental animals were not affected by the administration of either CCl_4_, G-M1 and PDG. However, a significant elevation of relative liver and spleen weight was seen at the end of the experimental procedure, indicating that CCl_4_ induced hypertrophy of these tissues. By contrast, G-M1 and PDG groups significantly reduced the elevated weight of liver (*P* < 0.05), suggesting their possible protective effects against liver injury after CCl_4_ induction. 

**Table 1:** Effects of PDG and G-M1 on body weight and relative organ weight in CCl_4_-intoxicated mice. Mean ± S.D.

Group	Body weight (g)	Relative weight (g/g body weight, %)		
		Heart	Liver	Spleen
Normal control	38.26 ± 2.15	0.51 ± 0.05	5.25 ± 0.31	0.42 ± 0.03
CCl_4_ control	37.25 ± 3.56	0.48 ± 0.06	6.98 ± 0.22 #	0.53 ± 0.09 #
G-M1 + CCl_4_	39.15 ± 3.33	0.47 ± 0.08	5.78 ± 0.12 *	0.44 ± 0.07
PDG + CCl_4_	40.05 ± 4.15	0.50 ± 0.04	6.23 ± 0.52 *	0.47 ± 0.11

* Significance *P* < 0.05, compared with CCl_4_ control; # Significance *P *< 0.05, compared with normal control group.

### 2.4. Effect of PDG and G-M1 on the Serum ALT and AST Levels

When the liver is injured by CCl_4_, membrane permeability of the liver parenchyma cells intensifies, and the activities of ALT and AST in serum increase sharply as a consequence. Serum amino-transferase activities have long been considered as sensitive indicators of hepatic injury [5,7].

**Figure 4:**
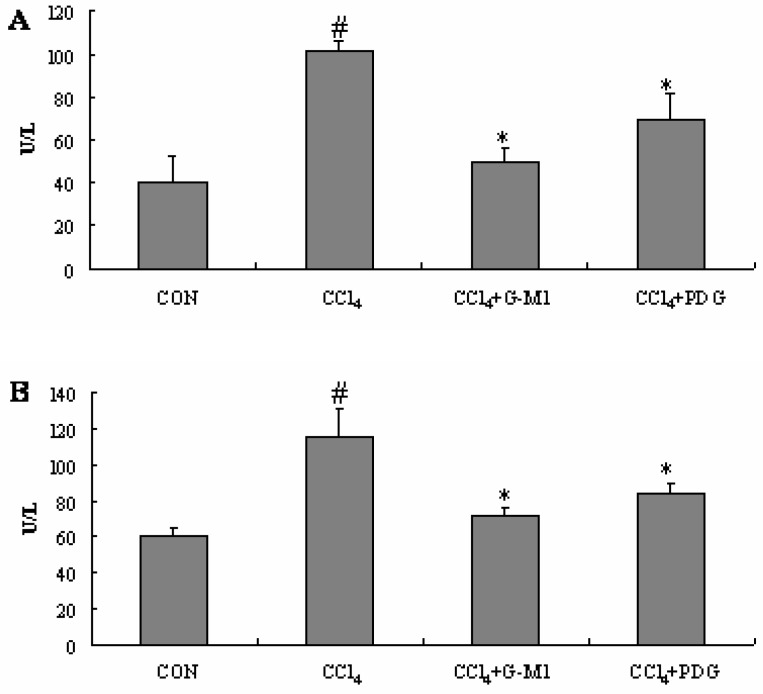
Effect of PDG and G-M1 on serum ALT and AST levels in CCl_4_-induced liver injury in mice. PDG and G-M1 were administrated for a period of 4 weeks at dose of 300 mg/kg and 30 mg/kg, respectively. Each value is mean ± S.D. for 12 mice in each group. * Significance *P* < 0.05, compared with CCl_4_ group. # Significance *P *< 0.05, compared with normal control group.

As shown in Figure 4, the serum levels of the hepatic enzymes AST and ALT were significantly elevated (*P* < 0.05) in the CCl_4_-treated mice. Compared with CCl_4_-treated mice (model group), treatment with PDG and G-M1 significantly prevented the elevation of these marker enzymes (*P* < 0.05). These results suggest the possibility that PDG and G-M1 can provide protection against liver injury after CCl_4_ induction.

### 2.5. Effect of PDG and G-M1 on the Level of SOD and MDA in Liver Homogenate

SOD is an effective defense enzyme that catalyses the dismutation of superoxide anions into hydrogen peroxide (H_2_O_2_). The activity of antioxidant enzyme SOD in liver homogenates was significantly decreased (*P* < 0.05) in liver injury model groups when compared to normal control. PDG and G-M1 exerted a beneficial effect on antioxidant enzyme since the SOD activity was found to be significantly increased in drug treated groups (*P* < 0.05).

Malondialdehyde (MDA), a secondary product of lipid peroxidation, is used as an indicator of tissue injury involving a series of chain reactions [26,27].

**Figure 5:**
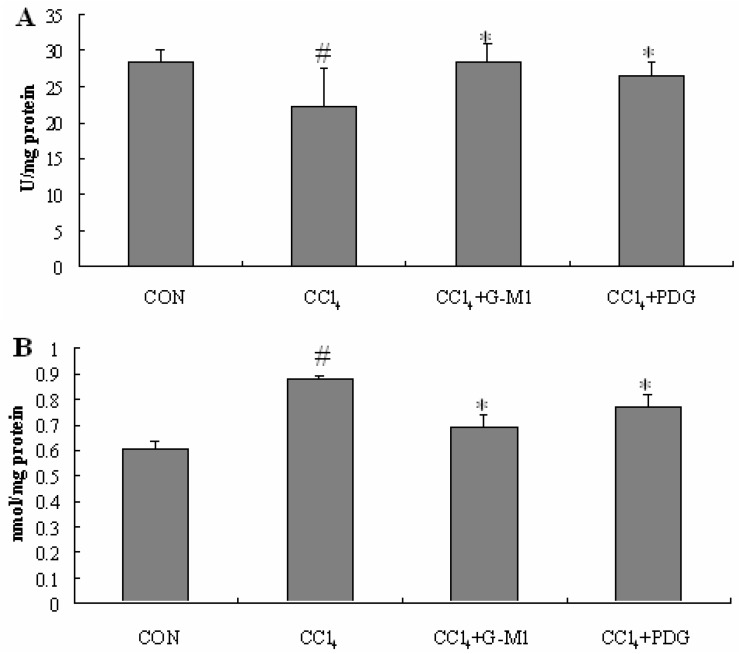
Effect of PDG and G-M1 on antioxidant enzyme (SOD) activities (**A**) and hepatic MDA level (**B**) in CCl_4_-induced liver injury in mice. PDG and G-M1 were administered for a period of 4 weeks at dose of 300 mg/kg and 30 mg/kg, respectively. * Significance *P* < 0.05, compared with CCl_4_ group. # Significance *P *< 0.05, compared with normal control group.

A significant increase in MDA level (*P* < 0.05), an indicator of lipid peroxidation, was found in the livers of CCl_4_-intoxicated mice relative to normal control. Treatment with PDG and G-M1 reversed this biochemical parameter significantly towards normal level (*P* < 0.05, Figure 5).

### 2.6. Histopathological Evaluation

**Figure 6:**
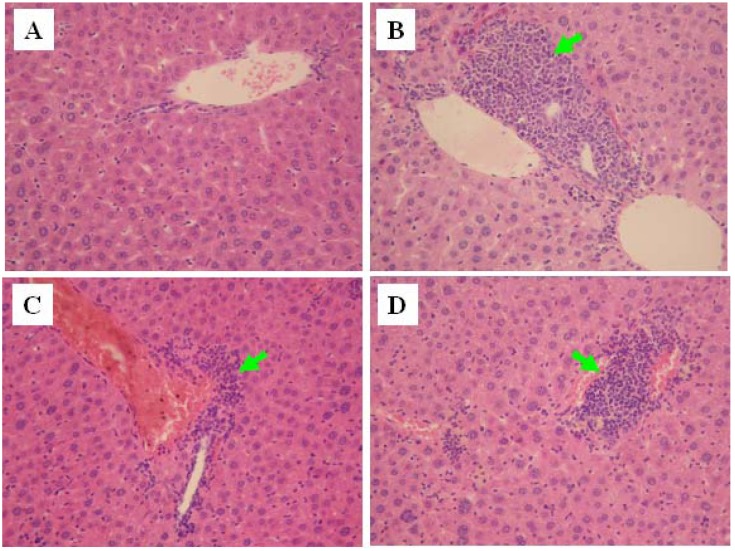
Histopathology of the liver (×100). (**A**) Normal control; (**B**) Treated with CCl_4_; (**C**) Treated with CCl_4_ + G-M1 (30 mg/kg/day); (**D**) Treated with CCl_4_ + PDG (300 mg/kg/day). The arrow represents leukocytes with infiltration.

Histopathologic examinations results showing hepatocyte necrosis and inflammatory cell infiltration, can be observed in Figure 6. In normal control animals, liver sections showed normal hepatic cells with well preserved cytoplasm, prominent nucleus and nucleolus, and central vein (Figure 6A The liver sections of animals treated with CCl_4_ showed a moderate degree of centrilobular necrosis, and mild degree of infiltration of leukocytes (Figure 6B). The histological observations also supported the results obtained from the serum enzyme assay. The histological pattern of the livers of the mice treated with G-M1 showed a lower degree of leukocyte infiltration and necrosis (Figure 6CD).

## 3. Experimental

### 3.1. Chemicals

CCl_4_ was purchased from Sigma Chemicals. The kits for determining AST, ALT, and SOD activities as well as MDA content were obtained from the Jiancheng Institute of Biotechnology, Nanjing, China. All other chemicals used were of analytical grade and obtained from Beijing Chemical Factory, Beijing, China. G-M1 used in this study was isolated and purified from *P. ginseng* roots in our laboratory by a series of chromatography procedures, and its structure was elucidated by comparison with spectral data [28]. Its purity was determined to be more than 98.5% by HPLC-UV analysis (Agilent 1100 series HPLC instrument).

### 3.2. Animals

Sixty male ICR mice (Experimental Animal Holding of Jilin University), 22 to 25 g, were housed individually in cages in a temperature-controlled room with a 12-hour light/dark cycle. After 1 week of acclimation, all mice were fasted for 16 h prior to blood/tissue sampling. All experiments were carried out in accordance with the guidelines for the Human Treatment of Animals set by the Association of Laboratory Animal Sciences and the Good Laboratory Practice Center.

### 3.3. Carbon Tetrachloride (CCl_4_)-Induced Chronic Liver Injury in Mice

For the chemical liver injury experiments, the animals were randomly divided into four groups each consisting of 12 mice. The experimental groups were as follows: Group I served as normal control. Groups II-IV were administered orally 5 mL/kg body weight of CCl_4_ (20% *v*/*v* in olive oil) once a week for a period of 8 weeks. From week-4 after CCl_4_ intoxication, Group II served as CCl_4_ control, Groups III-IV were gavaged with PDG and G-M1 daily for a period of 4 weeks at dose of 300 and 30 mg/kg, respectively. At the end of the experiment, animals were sacrificed by cervical dislocation. Blood was collected into non-heparinized capillary tubes and centrifuged (1,500 rpm, 10 min, 4 °C). Serum was aspirated and stored at −20 °C until assayed as described below. The liver was also removed and stored at −80 °C until use.

### 3.4. Measurement of Serum ALT and AST

Liver injury was assessed by estimating serum activities of alanine aminotransferase (ALT) and aspartate aminotransferase (AST) using commercially available test kits. The results were expressed as units/litre (U/L).

### 3.5. Measurement of SOD and MDA in Liver Homogenate

Liver samples were homogenized in Tris-HCl buffer (5 mM containing 2 mM of EDTA, pH 7.4) to give a 10% (*w*/*v*) liver homogenate. The homogenates were then centrifuged at 1,500 rpm for 15 min at 4 °C and the supernatants were used immediately for the determination of antioxidant status. The SOD activity, as well as the MDA level, was determined following the instructions on the kit. In brief, the assay for total SOD was based on its ability to inhibit the oxidation of oxyamine by the xanthine-xanthine oxidase system. The MDA content was determined by the thiobarbituric acid method. All samples were assayed in triplicates. The content of MDA was expressed as nanomole, while SOD activity was expressed as units per milligram protein (U/mg protein). Protein content of the homogenates was determined using a standard commercial kit provided by Beyotime Institute of Biotechnology (Shanghai, China).

### 3.6. Histopathological Evaluation

The livers were preserved in 100 mL/L neutral buffered formalin solution and processed routinely by embedding in paraffin. Tissue sections (4–5 μm) were stained with hematoxylin and eosin (H&E) stain, and observed under light microscope (Leica, Germany).

### 3.7. Statistical Analysis

All data are presented as mean ± standard deviations (S.D.). Statistical significance of the differences between groups was assessed by Student’s *t*-test. Calculations were performed using commercial software (GraphPad Software, San Diego, CA, USA). A level of *P* < 0.05 was taken as statistically significant.

## 4. Conclusions

To the best of our knowledge, snailase preparation of G-M1 from PDG has not been reported before. The present report highlighted this valuable biotransformation and investigated the process by reversed-phase HPLC. We have successfully achieved enzymatic conversion of PDG to G-M1 with treatment with snailase for 24 h at 50 °C and pH 4.5. We next evaluated the effects of PDG and G-M1 on serum ALT, AST and MDA levels, liver tissue antioxidant enzymes and liver histopathological changes in CC1_4_ treated mice. The results showed that PDG and G-M1 were effective in the prevention of CC1_4_-induced chronic liver injury, and the effects of G-M1 are more significant than that of PDG.
